# Special Issue on “Entomopathogenic Fungi: Ecology, Evolution, Adaptation”: An Editorial

**DOI:** 10.3390/microorganisms11061494

**Published:** 2023-06-04

**Authors:** Vadim Yu Kryukov, Viktor V. Glupov

**Affiliations:** Institute of Systematics and Ecology of Animals, Siberian Branch of Russian Academy of Sciences, Novosibirsk 630091, Russia

Entomopathogenic endophytic ascomycetes are the most widespread and commercially promising fungi and are used to solve many problems in basic and applied research in ecology, evolution, and agricultural sciences. For the latter, this topic is also relevant in terms of plant protection from phytophages and phytopathogens, plant growth and immunostimulation, and crop yield increase. Nevertheless, deeper research into relationships between fungi, insect hosts, plants, and other microorganisms is required in order to better understand the lifestyles of ascomycetes in ecosystems and improve their efficacy in practice. The species of the genera *Metarhizium* and *Beauveria* and certain species of *Cordyceps* sensu lato are the most widely studied entomopathogenic fungi. Nonetheless, many large territories of the world remain poorly investigated in regard to the diversity and patterns of spatial and temporal distributions of such species, even for these well-known genera. The study of pathogenesis, biochemical and molecular interactions with insects, and relationships with other organisms has also focused mainly on *Metarhizium* and *Beauveria* (reviewed in [1,2]). Undoubtedly, the mechanisms of many of these interactions have yet to be revealed, and new approaches of introduction into ecosystems will be proposed in the future. Moreover, species of other taxa may represent valuable resources and become practically useful in this field.

This Special Issue presents articles on the spatial distribution of entomopathogenic ascomycetes, their interactions with insects and plants, and specifics of the isolation and storage of the strains. Fernández-Bravo and coworkers [3] reported results on the habitat distribution of *Metarhizium* fungi in Switzerland. They showed that *Metarhizium* species are more abundant in grassland compared to forests and arable land, and *M. brunneum* is a predominant fungal species in this territory. The influence of soil physical and chemical properties on the distribution of *Metarhizium* species was also analyzed. Kazartsev and Lednev [4] studied the distribution and genetic diversity of *Beauveria* in boreal forests of European Russia and revealed differences in habitat distribution and haplotype diversity among *B. bassiana*, *B. pseudobassiana*, and *B. caledonica*. Among the articles on interactions of fungi with plants, Doherty and coauthors [5] reported the techniques of colonization of Carrizo Citrus by *Cordyceps fumosorosea* and the localization of this fungus in the plants. Tyurin et al. [6] investigated the distribution of *Metarhizium* and *Beauveria* fungi in potato agrosystems in Siberia, the frequency of potato colonization by the fungi, and the mycobiomes of potato roots and leaves. They demonstrated that *Metarhizium* and *Beauveria* may be common in the rhizosphere but scarce in the internal tissues of these plants. Ntsobi et al. [7] described the influences of *Clonostachys rosea* on composting and tomato growth and its protective effect against the red spider mite *Tetranychus urticae*. It was shown that the fungus promotes tomato seed germination and successfully colonizes the studied plants; however, there was no significant effect on the infestation of the plants by the mite. Sharma and coworkers [8] reviewed basic techniques for the isolation of entomopathogenic fungi and bacteria and listed the nutrient media appropriate for various taxa.

Several articles in this Special Issue focus on the roles of different metabolites in the adaptation of fungi to their hosts. In their review [9], Berestetskiy and Hu compared toxins from various ecological groups of fungi (entomopathogens, saprotrophs, phytopathogens, and endophytes) from the perspective of entomotoxicity. On the basis of a comprehensive literature analysis, the authors proposed that saprotrophic fungi are the most promising ecological group in the search for new insecticides. Kato and coworkers [10] researched the influence of the *Cordyceps militaris* metabolite cordycepin on the immune response of silkworm larvae and found that cordycepin can promote infections caused by other entomopathogenic fungi. Wellham and coauthors [11] identified decreasing levels of cordycepin during the subculture of *C. militaris*, and this phenomenon coincided with a decline in virulence and in sporulation on cadavers, as well as the underexpression of genes related to sexual development.

The papers presented herein contribute to knowledge about invertebrate pathology, fungal ecology and physiology, and plant protection. We thank all the authors of this Special Issue, “Entomopathogenic Fungi: Ecology, Evolution, and Adaptation” for their important contributions.
